# Gut Microbiota in Canine Idiopathic Epilepsy: Effects of Disease and Treatment

**DOI:** 10.3390/ani11113121

**Published:** 2021-10-31

**Authors:** Sylvia García-Belenguer, Laura Grasa, Olga Valero, Jorge Palacio, Isabel Luño, Belén Rosado

**Affiliations:** 1 Departamento de Patología Animal, Facultad de Veterinaria, Universidad de Zaragoza, Miguel Servet, 177, 50013 Zaragoza, Spain; olgavalerogimenez@hotmail.com (O.V.); jpalacio@unizar.es (J.P.); isalumu@hotmail.com (I.L.); belen@unizar.es (B.R.); 2 Departamento de Farmacología, Fisiología y Medicina Legal y Forense, Facultad de Veterinaria, Universidad de Zaragoza, Miguel Servet, 177, 50013 Zaragoza, Spain; lgralo@unizar.es; 3 Instituto de Investigación Sanitaria de Aragón (IIS), 50009 Zaragoza, Spain; 4 Instituto Agroalimentario de Aragón—IA2, Universidad de Zaragoza—CITA, 50009 Zaragoza, Spain

**Keywords:** microbiota, epilepsy, antiepileptic drugs, dogs

## Abstract

**Simple Summary:**

There is evidence that supports the existence of a gut-brain axis system through which bi-directional communication occurs between gut bacteria and the brain. Epilepsy is one of the most common neurological disorders in humans and dogs. The role of microbiota in epilepsy remains unknown but it has been suggested that it is a possible influence of gut bacteria in controlling seizures. The aim of this study was to investigate the changes in gut microbiota from dogs with idiopathic epilepsy and the possible effect of antiepileptic drugs on the modulation of the composition of this microbiota. In comparison with control dogs, drug-naive epileptic individuals showed a significantly reduced abundance of GABA and SCFAs-producing bacteria, as well as bacteria associated with reduced risk for brain disease. Moreover, the use of phenobarbital or imepitoin monotherapy during one month in epileptic dogs did not modify the gut microbiota composition. These results open up the possibility of studying probiotic interventions in epilepsy. Considering the phylogenetic and metabolic similarities in intestinal microbiome between humans and dogs, this study contributes to the understanding of epilepsy both in human and veterinary medicine.

**Abstract:**

Epilepsy is one of the most common neurological disorders in humans and dogs. The structure and composition of gut microbiome associated to this disorder has not yet been analyzed in depth but there is evidence that suggests a possible influence of gut bacteria in controlling seizures. The aim of this study was to investigate the changes in gut microbiota associated to canine idiopathic epilepsy (IE) and the possible influence of antiepileptic drugs (AEDs) on the modulation of this microbiota. Faecal microbiota composition was analyzed using sequencing of bacterial 16S rRNA gene in a group of healthy controls (*n* = 12) and a group of epileptic dogs both before (*n* = 10) and after a 30-day single treatment with phenobarbital or imepitoin (*n* = 9). Epileptic dogs showed significantly reduced abundance of GABA (*Pseudomonadales*, *Pseudomonadaceae, Pseudomonas* and *Pseudomona_graminis*) and SCFAs-producing bacteria (*Peptococcaceae, Ruminococcaceae* and *Anaerotruncus*) as well as bacteria associated with reduced risk for brain disease (*Prevotellaceae*) than control dogs. The administration of AEDs during 30 days did not modify the gut microbiota composition. These results are expected to contribute to the understanding of canine idiopathic epilepsy and open up the possibility of studying new therapeutic approaches for this disorder, including probiotic intervention to restore gut microbiota in epileptic individuals.

## 1. Introduction

Evidence of a bi-directional communication between the gastrointestinal tract, containing the gut microbiota, and the central nervous system (CNS) supports the existence of a gut-brain axis system [1].

Mutual information exchange between gut bacteria and the brain include neural, blood and immune-endocrine pathways [2]. In particular, gut microbiota was shown to affect health, behavior and cognitive functions in humans and animals by producing metabolites, hormones and immune factors [3,4]. It seems that a stable gut microbiota is essential for normal gut physiology and contributes to appropriate signaling along the brain-gut axis. Conversely, intestinal dysbiosis can adversely influence gut physiology, leading to inappropriate brain-gut axis signaling and associated consequences for CNS functions and disease states [5].

Some neuroactive molecules produced in the gut can cross the intestinal mucosal barrier and reach the bloodstream and then cross the Blood Brain Barrier (BBB) to reach the CNS [6,7]. For instance, *Escherichia coli* and *Pseudomonas* can synthetize γ-aminobutyric acid (GABA) (revised in Mazzolli and Pessione [1]), the major inhibitory neurotransmitter found in the CNS, which is able to cross the BBB [8]. Gut bacteria also produce short-chain fatty acids (SCFAs) such as butyrate, propionate, and acetate, which participate in mucus production and intestinal epithelial cell regeneration [9] and contribute to maintaining the integrity of the BBB. These SCFAs are the most abundant produced by anaerobic fermentation of dietary fibers in the intestine. Butyrate is a primary energy source for colonocytes and maintains intestinal homeostasis through anti-inflammatory actions [10]. SCFAs production by microbes plays an important role in decreasing intestinal pH, which prevents the growth of potentially pathogenic bacteria (revised by Bibbó et al. [11]). SCFAs can cross the BBB and reach the hypothalamus, where they regulate GABA, glutamate and glutamine levels and increase anorexigenic peptides expression [2,12]. The intestinal mucosal barrier and BBB permeability are affected by several factors, including stress, diet and gut microbiota [6,7].

Epilepsy is one of the most common neurological disorders in humans and dogs. The prevalence of epilepsy in humans has been estimated to be 0.64% in the general population [13,14]. The true prevalence of epilepsy in dogs is unknown but studies carried out in the United Kingdom estimated a prevalence of 0.62–0.82% in the general dog population [15,16]. Epilepsies of unknown etiology show the highest prevalence both in humans (49.4% of epileptic population) [13] and dogs (48–54.9% of epileptic dogs) [17,18]. Drug-refractory epilepsy individuals represent 30–40% of patients in both human and veterinary medicine [19,20]. These cases require a more in-depth study of possible predisposing and risk factors, including the structure and composition of gut microbiome.

The role of microbiota in epilepsy remains unknown, but there are several works that suggest a possible influence of the gut bacteria in controlling seizures [21]. Olson et al. [22] demonstrated that a ketogenic diet (KD) alters the gut microbiota across two seizure mouse models for refractory epilepsy and that changes in microbiota are necessary and sufficient for conferring seizures protection. This study also revealed diet- and microbiota-dependent regulation of hippocampal GABA and glutamate levels in mice, in accordance with prevailing theories that consider GABA contributes to the antiseizure effects of the KD [23]. All of these findings are relevant, since an imbalance between the inhibitory (GABA) and the excitatory (glutamate) neurotransmission, in favor of the latter, typically is viewed as the basic pathophysiologic mechanism of seizures [24].

On the other hand, it has also been speculated that intestinal dysbiosis may be an important factor in the development and/or severity of epilepsy and that immune-stimulation by the microbiota could provide an alternative strategy for treatment of inflammation-related diseases such as epilepsy [25]. In humans, a probiotic treatment was shown to reduce seizure frequency by 50% or more in 28.9% of studied patients with drug-resistant epilepsy and this was associated with a significant improvement in their quality of life [26].

In dogs, alterations in the composition of the gut microbiota were associated with gastrointestinal dysfunctions [27] and obesity [28], and more recently, with neurological diseases such as meningoencephalomyelitis of unknown origin [29] and behavioral problems such as aggression and phobic disorders [30,31]. There are still very few studies on the role of microbiota in epileptic dogs. Muñana et al. [32] evaluated *Lactobacillus* populations in dogs with IE compared to healthy dogs by obtaining faecal samples from 13 pairs of dogs, consisting of a drug-naive epileptic dog and a healthy dog from the same household and maintained on the same diet. They did not identify any difference in large-scale microbial patterns or (relative or absolute) abundance of *Lactobacillus* species between groups. Pilla et al. [33] studied faecal microbiota in epileptic dogs fed with a medium chain triglyceride ketogenic diet (MCT-KD), a diet with neuroprotective properties that has been clinically demonstrated to reduce the occurrence of seizures in dogs [34,35,36]. The administration of this MCT-KD resulted in a significant increase in the species richness of bacterial communities within samples (alpha-diversity), but no differences were found in phylogenetical diversity between samples (beta-diversity). In addition, a bacteria of the genus 5-7N15 of family *Bacteroidaceae* was moreover a potential biomarker associated with MCT-KD [33]. Considering these results, it is necessary to further investigate the role of gut microbiota in canine epilepsy.

The dog provides unique features as a spontaneous model of neurological and behavioral diseases and gut-brain axis function [37]. Domestication has led dogs and humans to share environment and diet characteristics, and both suffer from similar neurological and behavioral diseases, such as epilepsy, dementia, compulsive disorders or anxiety [38,39,40]. In particular, phylogenetic and metabolic similarities in intestinal microbiome were demonstrated between humans and dogs [41]. Canine faecal samples reliably present most of the relevant taxa, unlike humans, in which most significant taxa are closely associated with the mucosa [4]. This fact might facilitate the study of the gut microbiota of dogs through the collection of faecal samples.

The aim of this study was to investigate the changes in the gut microbiota from dogs with idiopathic epilepsy (IE) and the possible effect of antiepileptic drugs (AEDs) on the modulation of the composition of this microbiota. To this end, the phylogenetic composition and structure of the faecal microbiota was profiled and subsequently compared between a group of healthy controls and a group of drug-naive epileptic dogs. Then, the gut microbiota composition of epileptic dogs was compared before and after a 30-day treatment with a single AED (phenobarbital or imepitoin).

## 2. Materials and Methods

### 2.1. Animals and Procedures

The study population consisted of 10 epileptic dogs (E group) from different breeds and 12 healthy beagles (C group). The epileptic dogs were submitted to the neurology service of a veterinary teaching hospital (Hospital Veterinario de la Universidad de Zaragoza) owing to a problem of seizures. Diagnosis of IE was made according to the tier I confidence level criteria from International Veterinary Epilepsy Task Force (IVETF), which describes a history of two or more unprovoked epileptic seizures occurring at least 24 h apart, age at epileptic seizure onset of between 6 months and 6 years, unremarkable inter-ictal physical and neurological examination and no clinically significant abnormalities on minimum data base blood test (complete blood cell count and serum biochemistry including sodium, potassium, chloride, calcium, phosphate, alanine aminotransferase, alkaline phosphatase, total bilirubin, urea, creatinine, total protein, albumin, glucose, cholesterol, triglycerides and fasting bile acids and/or ammonia) and urinalysis (specific gravity, protein, glucose, pH, and sediment cytology) [42]. All of the animals were fed with a commercial maintenance food (composition characteristics range: 22–30% crude protein, 7–18% crude fat, 5.3–10.5% crude ash, 1.3–10% fiber) and were not receiving pharmacological nor nutraceutical treatments for epilepsy or other medical conditions at the moment of the enrollment.

The control dogs were owned by the University of Zaragoza (Faculty of Veterinary) for research purposes. These beagles had daily contact with humans, both caretakers and students. Moreover, they were walked by organized groups of students every weekday in a large fenced enclosure located at the Faculty facilities where they could freely run and play. At the moment of the study, they were all healthy and lacked any history of seizures or other medical conditions and were fed with the same commercial maintenance food (25% crude protein, 14% crude fat, 6.1% crude ash, 1.3% fiber).

Both epileptic and control dogs were properly dewormed and vaccinated. The stool study of parasites was negative at the time of inclusion in the study.

Faecal samples (1–3 g) were collected directly from the rectal ampoule with sterile gloves both in epileptic dogs before treatment (E, *n* = 10) and healthy controls (C, *n* = 12), and immediately frozen at −80 °C to fix bacterial growth and preserve DNA content. In the case of nine epileptic dogs, samples were also collected after one month on a stable dose of a single-AED treatment (Ed, *n* = 9). Of these, four were being treated with phenobarbital and five with imepitoin. During the period between the two sampling moments, no changes on handling or feeding were conducted in these animals other than the introduction of the AED. All of them showed a good clinical response for at least the following 3 months after concluding the study.

#### Ethics Statement

Before enrollment, the owners of epileptic dogs were informed about the study and procedures, and they were asked for permission to take the faecal sample. They were given the opportunity to ask any questions and confirm or decline participation. All procedures were carried out under Project License PI27/18 approved by the Ethic Committee for Animal Experiments from the University of Zaragoza. The care and use of control dogs were performed accordingly to the Spanish Policy for Animal Protection RD53/2013 which meets the European Union Directive 2010/63 on the protection of animals used for experimental and other scientific purposes.

### 2.2. Faecal Microbiota Analysis

Bacterial DNA was extracted from faecal samples using the NZY Soil gDNA Isolation kit (NZYTech, Lisboa, Portugal) following the manufacturer’s instructions and minor modifications. Stool samples (120–180 mg) were mixed with 700 µL NSL1 buffer in NZYSpin Soil Bead Tubes and processed by using the Precellys^®^ 24 homogenizer (Bertin Instruments, Montigny-le-Bretonneux, France) for 2 × 30 s at 6500 rpm and 10 s delay between cycles. Once DNA was extracted, the concentration of the DNA was measured with a Qubit^®^ 4.0 fluorometer (Invitrogen, Thermo Fisher Scientific, MA, USA). DNA purity was assessed by measuring the A260/A280 with a NanoDrop^®^ ND-1000 Spectrophotometer V3.0.1 (Thermo Scientific, MA, USA) and monitored on 1% agarose gels.

### 2.3. Sequencing of Bacterial 16S rRNA Gene

According to the concentration, DNA was diluted to 1 ng/μL using sterile water. The 16S rRNA gene of the V4 region was amplified using a specific primer (515F-806R) [43] with a barcode. All PCR reactions were carried out with Phusion^®^ High-Fidelity PCR Master Mix (New England Biolabs, UK). The same volume of 1 × loading buffer (contained SYBR green) was mixed with PCR products and amplicons were detected by electrophoresis on 2% agarose gel. Samples with a bright main strip between 400–450 bp were chosen for further experiments. PCR products were mixed in equidensity ratios. Then, the mixture of PCR products was purified with the Qiagen Gel Extraction Kit (Qiagen, Germany).

Sequencing libraries were generated using the NEBNext Ultra DNA Library Pre^®^ Kit for Illumina^®^ (New England Biolabs, Ipswich, MA, USA), following the manufacturer’s recommendations and index codes were added. The library quality was assessed on the Qubit 2.0 Fluorometer and Agilent Bioanalyzer 2100 system (Agilent, Santa Clara, CA, USA). Later, the library was sequenced on an Illumina MiSeq platform and 250 bp paired-end reads were generated. A demultiplexing process to sort the sequenced reads into separate files was carried out at the end. Paired-end reads were assigned to samples based on their unique barcode and truncated by cutting off the barcode and primer sequence. The data of the sequences are available in NCBI Sequence Read Archive (SRA), BioProject ID PRJNA746550.

### 2.4. Bioinformatics

#### 2.4.1. Sequencing Data Processing

Paired-end reads were merged using FLASH V1.2.7 [44] and the splicing sequences were called raw tags. Quality filtering on the raw tags was performed under specific filtering conditions to obtain the high-quality clean tags [45] according to the QIIME (Version 1.7.0) [46] quality controlled process. The tags were compared with the reference database (Gold database) using the UCHIME algorithm [47] to detect chimera sequences; then the chimera sequences were removed [48] and the effective tags obtained.

#### 2.4.2. OTU Cluster and Taxonomic Annotation

Sequence analyses were performed by Uparse software v7.0.1001 [49] using all the effective tags. Sequences with ≥97% similarity were assigned to the same Operational Taxonomic Units (OTUs). The representative sequence for each OTU was screened for further annotation. For each representative sequence, Mothur software was performed against the SSUrRNA database of the SILVA Database [50] for species annotation at each taxonomic rank (Threshold: 0.8~1) [51]. To obtain the phylogenetic relationship of all OTUs representative sequences, the MUSCLE (Version 3.8.31) [52] was used. OTUs abundance information was normalized using a standard of sequence numbers corresponding to the sample with the least sequences. Subsequent analysis of alpha diversity and beta diversity were all performed based on this output normalized data.

#### 2.4.3. Alpha and Beta Diversity

Alpha diversity was applied to analyze complexity of biodiversity for a sample through two indices, Observed species and Shannon. Both indices in our samples were calculated with QIIME (Version 1.7.0) and displayed with R software (Version 2.15.3).

In order to study the beta diversity, a multivariate cluster analysis based on the Bray–Curtis distance was performed using QIIME software (Version 1.7.0) and a Non-metric Multidimensional Scaling (NMDS) was represented to show the dissimilarity between groups. Anosim was performed by R software (Vegan package). A t-test and drawing were conducted by R software. The t-test was performed to determine species with significant variation between groups (*p* value < 0.05) at various taxon ranks including phylum, class, order, family, genus, and species. This analysis captured those species whose abundance varied significantly among groups, meanwhile, the distribution of these variant species among the groups was also obtained. By comparing the within group variation and variation among groups, whether the variation of the community structure among different groups is significant can be determined.

## 3. Results and Discussion

### 3.1. Demographic Information

The epileptic (E) group consisted of 10 dogs (6 males and 4 females) aged between 2–6 years old (mean ± SD, 4.4 y ± 2.1) and weighed between 6–68 kg (mean ± SD, 21.6 kg ± 18.4). This group included dogs of different breeds: Border collie (1), French Bulldog (1), Golden retriever (1), Saint Bernard (1), Spitz (1), and 5 crossbred dogs. The control (C) group consisted of 12 healthy beagles (7 females and 5 males) aged between 2–9 years old (4.2 years ± 2.9) and weighed between 11.2–17.0 (14.9 kg ± 1.8). There were no significative differences between groups neither in age nor in weight.

### 3.2. Gut Microbiota Relative Abundance in the Studied Dogs

The minimum and maximum number of sequences present in the samples were 119,185 and 219,559, the mean being 204,529 sequences. The maximum and minimum length of the unique sequences was 441 and 51 nts, respectively.

**Figure 1:**
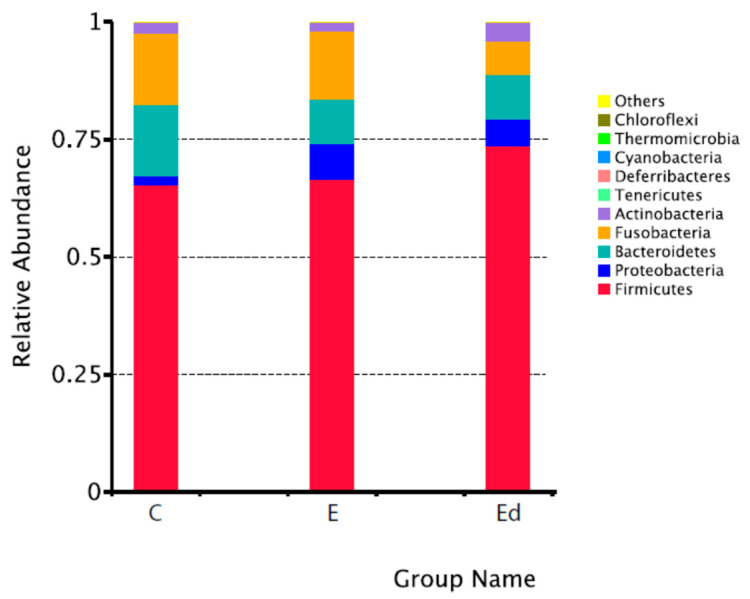
Relative abundance of the top ten phyla in healthy controls (C), drug-naive epileptic dogs (E) and treated epileptic dogs with an AED (Ed).

Table 1 shows the relative abundance of the ten predominant bacteria groups within each of the different taxa from phyla to genus both in healthy controls and epileptic dogs (before and after treatment). There were no significant differences between healthy and epileptic dogs in the distribution of these dominant bacteria taxa. In addition, Figure 1 graphically represents the top ten phyla in the studied groups of dogs. In agreement with previous studies in canine species, Firmicutes, Bacteriodetes, Fusobacteria, Proteobacteria and Actinobacteria were the predominant bacterial phyla in analyzed faecal samples, although proportions vary among studies. In particular, we found a higher proportion of Firmicutes (65%) when compared to other studies (47.7% in Suchodolski et al. [53]; 14–28% in Milddelbos et al. [54]; 23.6% in Alessandri et al. [55]) but very similar to Mondo et al. [31] (68%). This variability among studies may be due to laboratory methodology, individual characteristics, living environment [56] and even geographical location.

In accordance with previous studies, the most prevalent bacterial class and order were Clostridia and Clostridiales, respectively [28,57], dominated by the genus *Peptoclostridium* both in healthy and epileptic dogs. *Blautia* and *Ruminococcus* genera, also belonging to the Clostridiales order, were likewise significantly represented in control and epileptic groups. In addition to Clostridia, additional prevalent bacterial classes and orders were Fusobacteria and Fusobacteriales, and Bacteroidia and Bacteroidales, respectively. At the genus level, a significant presence of *Fusobacterium*, *Bacteroides, Prevotella*_9 (belonging to the Bacteroidetes phylum) and *Megamonas* (belonging to the Firmicutes phylum) were also detected. In particular, *Fusobacterium* was the dominant genus from Fusobacteriales, as previously described in different canine breeds [55]. Moreover, Bacteroides and *Prevotella*_9 genera were linked to a vegetarian diet and to the transition from a carnivorous diet to an omnivorous one in the dog [55]. Finally, *Streptococcus* and *Lactobacillus* genera belonging to the *Lactobacillales* order (from *Bacilli* class), were also between the top ten genera in the analyzed faecal microbiota both in healthy and epileptic dogs. Ingestion of *Lactobacillus* strain was associated to regulation of emotional behavior and central GABA receptor expression [58].

In the present study, breed differences were not specifically addressed beyond the difference between beagles (controls) and epileptic dogs, which belonged to different breeds. Alessandri et al. [55] did not find differences in the gut microbiota across dog breeds, but other authors have detected some differences, for instance, in the abundance of Fusobateria, which was higher in Maltese compared to Poodle or miniature Schnauzer [59,60].

On the contrary, Alessandri et al. [55] found significant differences by age and diet, as well as by human cohabitation, in comparison with wolves. In particular, they found a higher relative abundance of Bacteroidales in puppies (0–8 months old), *Phascolarctobacterium* in juniors (9–24 months old), *Fusobacterium* in adults (25–96 months old) and *Roseburia* in seniors (>96 months old), and a significant reduction in the abundance of *Biffidobacterium* genus in adults and seniors, similar to that observed during ageing in humans [55]. You and Kim [59] also found that *Fusobacterium perfoetens* was significantly more abundant in dogs 6–10 year-old than in 0.5–1 year-old ones. In the present study, all dogs were young adults (except two 9-year old control dogs) and there were no significant differences by age between groups.

Apart from breed and age, diet is considered a major factor shaping the composition of the intestinal microbial community both in humans [21] and animals, including dogs [55,61]. In this study, control dogs (but not epileptic ones) were housed in the same environment (university facilities) and fed with the same diet, as a part of a common protocol of handling. The use of a single-breed colony of dogs instead of a group of matched pet healthy dogs poses a limitation of this study, as epileptic dogs were fed with different commercial foods. Moreover, even the beagles have daily contact with humans; their living environment might differ from most owned dogs. Despite all of these limitations, we believe the fact of having a homogeneous control group in terms of breed, age, diet and housing conditions might represent an advantage in terms of providing a reference for “normal” gut microbiota. Nevertheless, in the absence of a standard pattern of normality for canine gut microbiota, comparison of healthy and epileptic dogs, which is discussed below, should be treated with caution and further research is needed to validate the present results.

Finally, the same protocol for sample extraction as well as laboratory processing was used in control and epileptic dogs, therefore reducing possible methodological-related differences between groups.

### 3.3. Gut Microbiota Differences between Healthy and Drug-Naive Epileptic Dogs: Effect of Disease

**Figure 2:**
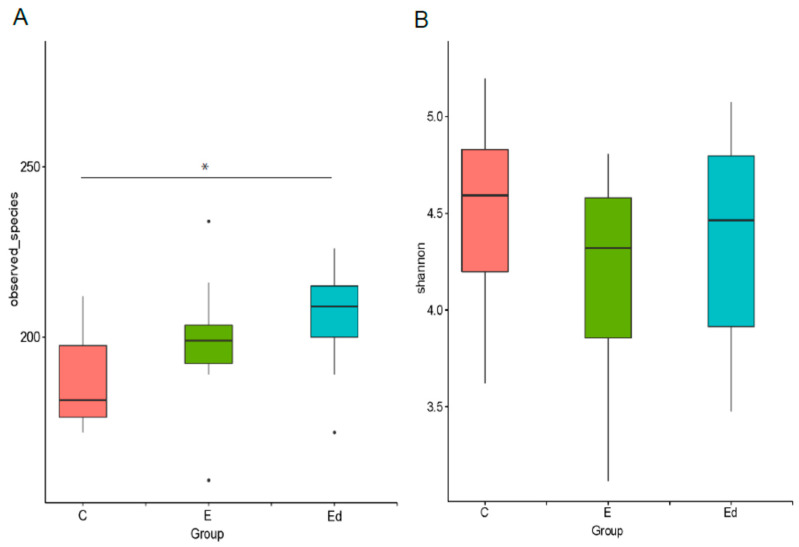
Alpha diversity of the faecal bacterial communities of controls (C), drug-naive epileptic dogs (E) and epileptic dogs after treatment with an AED (Ed). Boxplots show the alpha diversity measures computed with non-phylogenetic metrics: (**A**) Observed species index and (**B**) Shannon diversity index. Asterisk denotes the statistical difference between C y Ed (*p* < 0.05).

To estimate and compare the alpha diversity (intra-individual diversity) of the faecal microbial community derived from both healthy and epileptic dogs (before and after AED treatment), the Observed species (Figure 2A) and the Shannon biodiversity index (Figure 2B) was used. According to these metrics, there were no differences in alpha diversity between control and drug-naive epileptic groups (mean ± SD, Observed species: 186.5 ± 15.5, Shannon index: 4.5 ± 0.5, and Observed species: 198.6 ± 19.5, Shannon index: 4.2 ± 0.5, respectively). However, the Observed species metric highlighted a significant difference between the control and the treated epileptic group, that showed the higher number of different taxa observed (mean ± SD, Observed species: 205.3 ± 16.7, *p* < 0.05, Kruskal–Wallis test). The statistically significant difference in the alpha diversity between the control and the treated epileptic group might be accounted for the AED treatment effect but this seems unlikely considering the absence of differences between drug-naive and treated epileptic groups. Further studies including a large number of animals should be necessary to elucidate the biological significance of this finding. Alpha diversity indices from faecal samples in dogs differ among studies, both in normal (Observed species: 136 in Handl et al. [57]; 837, range 586–1120 in Bresciani et al. [62]; 759 ± 78 in Schmidt et al. [63]; 454.8 ± 118.3 in Mondo et al. [31]) and epileptic dogs (532, range 356–749 in Pilla et al. [33]). These differences could be attributed to previously discussed factors such as laboratory methodology, individual characteristics and living environment [56]. Despite greater biodiversity has been attributed to health in several studies in humans [64,65] and dogs, mainly in relation to gastrointestinal diseases [66], this attribution is not so clear in other pathological conditions. Thus, in human patients with drug-resistant epilepsy it has been described as an increase in alpha diversity compared to patients with drug-sensitive epilepsy [21]. The same results were found in children with drug-resistant epilepsy compared to healthy sibling controls; moreover, treatment with a ketogenic diet showed a tendency to decrease the alpha diversity indices in these children [67]. On the other hand, no significant differences were detected in alpha diversity between healthy and meningoencephalitis of unknown origin diagnosed dogs [29] or between aggressive and non-aggressive dogs [30].

**Figure 3:**
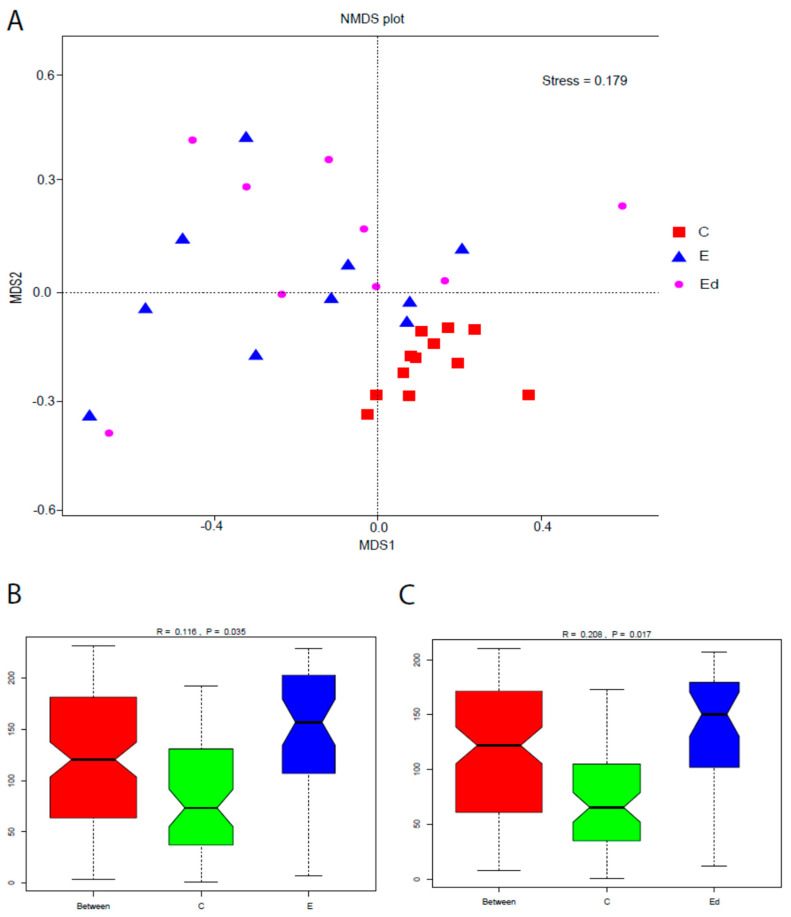
Beta diversity of the faecal bacterial communities of controls (C), drug-naive epileptic dogs (E) and epileptic dogs after treatment with an AED (Ed). (**A**) Non-metric Multidimensional Scaling (NMDS) plots show that C and E were are distinct clusters. (**B**,**C**) Analysis of similarity (Anosim) evidenced significant differences between C and E dogs and between C and Ed, but not between epileptic dogs before (E) and after treatment (Ed).

To assess the overall difference of bacterial community (beta diversity) between samples from controls, drug-naive epileptic dogs and epileptic with single-AED treatment dogs, Non-metric Multidimensional Scaling (NMDS) was performed. The NMDS of the inter-sample variation plots (Figure 3A) evidenced that control and epileptic dogs were clearly asymmetric and distant populations (R = 0.116; *p* = 0.035). However, the gut microbiota of epileptic dogs seems to be similar to that of epileptic dogs after treatment with an AED. Analysis of similarity (Anosim) (Figure 3B) evidenced significant differences between C and E dogs (Anosim R = 0.116; *p* = 0.035) and between C and Ed dogs (Anosim R = 0.208; *p* = 0.017) but not between E and Ed dogs (Anosim R = −0.021; *p* = 0.607).

**Figure 4:**
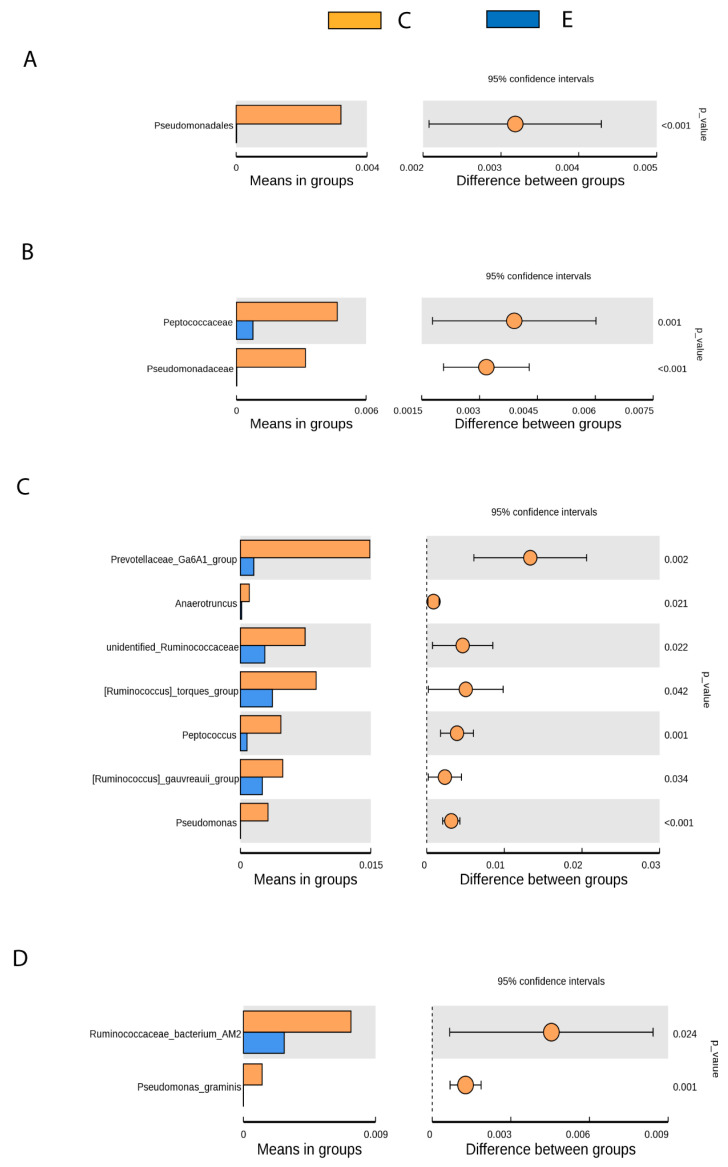
T-test results corresponding to significant differences in the distribution of the relative abundance of bacteria between controls (C) and drug-naive epileptic dogs (E) at different levels: order (**A**), family (**B**), Genus (**C**) and Species (**D**).

In particular, the *t*-test (Figure 4) revealed significant differences between healthy and drug-naive epileptic dogs in faecal samples for several bacteria not belonging to the predominant bacteria taxa but to the minority ones. Thus, it was found that epileptic dogs compared to healthy dogs showed significantly reduced abundance of:

1. *Pseudomonadales* (Order), *Pseudomonadaceae* (Family), *Pseudomonas* (Genus), *Pseudomona_graminis* (Species) (*p* < 0.001).
2. *Prevotellaceae Ga6A1* group (Genus) (*p* < 0.05).
3. *Peptococcaceae* (Family), *Anaerotruncus, unidentified Ruminococaceae, Ruminococcus torques* group, *Peptococcus y Ruminococcus gauvreauii* group (Genus), *Ruminococcaceae bacterium_AM2* (Species) (*p* < 0.05).

The differences between healthy and epileptic dogs related to *Pseudomonas* level seem of particular interest since they are bacteria that produce GABA from glutamate [1]. GABA transporters are mostly in the CNS [68] and different studies suggest that GABA produced in the gut should be able to cross BBB, although in small amounts [8]. Therefore, a decrease in this bacteria group could affect availability of GABA, an essential neurotransmitter in seizures control. In fact, it has been suggested that GABA contributes to the antiseizure effects of the KD [23]. Conversely, GABA could have an effect on the physiology of *Pseudomonas*, and specific receptors for sensing GABA have been found in *Pseudomonadales* [1,69]. For instance, GABA increases the cytotoxicity and virulence of *Pseudomona aeruginosa* [70,71]. These findings evidence the importance of the balance between *Pseudomonadales* and GABA.

In particular, the least represented *Pseudomonas* species in the epileptic group was *Pseudomona graminis*. One strain of this species is being studied in the food industry as a biocontrol agent against foodborne pathogens on fresh-cut fruit [72]. However, to the knowledge of the authors, there are not published studies relating this species to any medical condition in humans or dogs.

Jeffery et al. [29] found that *Prevotellaceae* were significantly less abundant in dogs showing meningoencephalomyelitis of unknown origin compared with controls, providing evidence that high abundance of this bacteria in the gut was associated with reduced risk for developing immune-mediated brain disease. These results align with those found in the present study, where epileptic dogs showed a significant reduction in Prevotellaceae in comparison with healthy individuals. Therefore, the role of this bacteria genus in conferring seizure protection should be further explored in epileptic dogs as well as in other neurological conditions.

*Ruminococcaceae* and *Peptococcaceae* affiliated to the order Clostridiales, are SCFAs-producing bacteria [10]. SCFAs can cross the BBB and reach the hypothalamus, where they can regulate GABA, glutamate and glutamine levels and increase anorexigenic peptides expression (revised in Gómez-Eguilaz et al. [2]). Similar to our results, *Ruminococcus* together with other bacteria were found notably decreased in epileptic children in comparison with healthy controls [73]. Reduced abundance of *Ruminococcus* was also found in individuals with bipolar depression compared to healthy controls. When these depressed patients were treated with probiotics supplements in addition to standard therapy, the *Ruminococcus gauvreauii group* appeared more abundant and diversity was higher in these patients than in the placebo group, showing an overall beneficial effect of clinical treatment by helping to balance microbiota composition [74].

*Anaerotruncus* genus, which was also reduced in epileptic dogs compared to controls, was recently associated, along with *Clostridium sensu stricto*, with protection from hepatocellular cancer in men with cirrhosis [75]. Another study found that dried plum feed supplement in birds can mitigate heat stress by changes in microbiota, increasing bacteria such as *Anaerotruncus*, among others [76]. On the other hand, a high saturated fat/low fiber diet was associated with a greater sequence abundance of the *Anaerotruncus* genus, a butyrate producer associated with obesity [77]. The possible association of *Anaerotruncus* and epilepsy remains to be explored.

Despite the higher relative abundance of *Lactobacillales* (order)*, Lactobacillaceae* (family) and *Lactobacillus* (genus) in epileptic than in control dogs (Table 1), we did not find significant differences between groups. Similarly, Muñana et al. [32] did not identify any difference in large-scale microbial patterns of relative or absolute abundance of *Lactobacillus* species in drug-naive epileptic dogs compared to healthy matched dogs. Interestingly, *Lactobacillus* was shown to be relatively more abundant in phobic [31] and aggressive [30] dogs than in normally behaved control dogs. The absence of significant differences in abundance of *Lactobacillus* between healthy and epileptic dogs does not mean that the administration of some strains could not have a potential beneficial effect in mitigating canine epilepsy or anxiety, but this might deserve further investigation. In this line, the administration of *Lactobacillus rahmnosus* in mice induced region-dependent alterations in GABA_B1b_ mRNA in the brain and reduced stress-induced corticosterone as well as anxiety and depression-related behavior. This neurochemical and behavioral effect was possibly mediated by the vagus nerve [58]. Moreover, GABA production was speculated as the key factor in the ability of *Lactobacillus helveticus* to reduce anxiety-like behaviors in rats and human subjects [71].

### 3.4. Gut Microbiota Changes after Introduction of AEDs: Effect of Treatment

Alpha diversity metrics, as described by Observed species and Shannon indices, were not significantly different before and after treatment in the epileptic dogs (Figure 2). Moreover, as it was shown in the beta diversity analysis, differences in gut microbiota between healthy and epileptic dogs were maintained regardless of treatment with a single AED (Figure 3). That is, treatment with phenobarbital or imepitoin during one month did not affect the composition of gut microbiota in epileptic dogs. Muñana et al. [32] observed that *Lactobacillus* in culture were not killed by exposure to phenobarbital, potassium bromide, zonisamide or levetiracetam, suggesting that AED drug administration in dogs is less likely to be a confounding factor in future studies evaluating the role of *Lactobacillus* in canine epilepsy. Our results also support the idea that exposure to imepitoin or phenobarbital does not have a significant influence on gut microbiota composition, under in vivo conditions. Nevertheless, more specific studies are needed to investigate the significance of interactions between AEDs and microbiome for the treatment of epilepsy, as it occurs in human medicine [21].

## 4. Conclusions

This is the first study that profiles the whole phylogenetic composition and structure of faecal microbiota in healthy and epileptic dogs both before and after treatment with an AED. Firmicutes, Bacteriodetes, Fusobacteria, Proteobacteria and Actinobacteria were the predominant bacterial phyla in both healthy and epileptic dogs. However, significant differences in microbiome patterns emerged between the groups of study at several levels. In comparison with healthy laboratory beagles, drug-naive epileptic individuals showed a significantly reduced abundance of GABA (*Pseudomonadales*, *Pseudomonadaceae, Pseudomonas* and *Pseudomona_graminis*) and SCFAs-producing bacteria (*Peptococcaceae, Ruminococcaceae* and *Anaerotruncus*), as well as bacteria associated with reduced risk for brain disease (*Prevotellaceae Ga6A1 group*). Although this study is not exempt from limitations derived from the number of animals and the differences inherent to the healthy control group in relation to the epileptic group, these findings are of great interest since GABA is an essential neurotransmitter in seizures control and SCFAs can regulate GABA levels at the CNS. Possible adoption of probiotic interventions aimed at restoring reduced bacteria groups in canine IE might deserve future investigation. On the other hand, the use of phenobarbital or imepitoin monotherapy during one month in epileptic dogs did not modify the gut microbiota composition, but more studies including a higher number of dogs are needed to investigate the interactions between different AEDs and gut microbiome in IE.

## Figures and Tables

**Table 1:** Relative abundance (percentage in decreasing order) of top ten bacteria groups belonging to different taxa from phyla to genus in healthy controls (C, *n* = 12), drug-naïve epileptic dogs (E, *n* = 10) and treated epileptic dogs with an AED (Ed, *n* = 9).

Philum	Class	Order	Family	Genus
*Firmicutes*	*Clostridia*	*Clostridiales*	*Lachnospiraceae*	*Peptoclostridium*
C: 65.4 ± 16.7%	C: 54.5 ± 15.4%	C: 54.5 ± 15.4%	C: 26.5 ± 10.4%	C: 21.8 ± 7.9%
E: 66.6 ± 16.1%	E: 50.0 ± 13.6%	E: 50.0 ± 13.6%	E: 26.6 ± 11.6%	E: 17.9 ± 8.0%
Ed: 73.7 ± 14.3%	Ed: 54.0 ± 11.3%	Ed: 54.0 ± 11.3%	Ed: 29.0 ± 8.0%	Ed: 19.3 ± 9.6%
*Bacteroidetes*	*Bacteroidia*	*Bacteroidales*	*Peptostreptococcaceae*	*Fusobacterium*
C: 15.3 ± 9.5%	C: 15.3 ± 9.5%	C: 15.3 ± 9.5%	C: 22.5 ± 7.9%	C: 12.8 ± 8.2%
E: 9.5 ± 7.3%	E: 9.5 ± 7.3%	E: 9.5 ± 7.3%	E: 19.5 ± 7.6%	E: 12.5 ± 8.4%
Ed: 9.5 ± 8.2%	Ed: 9.5 ± 8.2%	Ed: 9.5 ± 8.2%	Ed: 20.6 ± 9.2%	Ed: 5.7 ± 5.5%
*Fusobacteria*	*Fusobacteriia*	*Fusobacteriales*	*Fusobacteriaceae*	*Blautia*
C: 15.2 ± 9.1%	C: 15.2 ± 9.2%	C: 15.2 ± 9.2%	C: 12.8 ± 8.2%	C: 12.2 ± 5.5%
E: 14.5 ± 10.0%	E: 14.5 ± 10.0%	E: 14.5 ± 10.0%	E: 12.5 ± 8.4%	E: 12.5 ± 7.1%
Ed: 7.0 ± 7.1%	Ed: 7.0 ± 7.1%	Ed: 7.0 ± 7.1%	Ed: 5.7 ± 5.5%	Ed: 13.8 ± 4.3%
*Proteobacteria*	*Erysipelotrichia*	*Erysipelotrichiales*	*Bacteroidaceae*	*Bacteroides*
C: 1.8 ± 0.8%	C: 6.6 ± 3.3%	C: 6.6 ± 3.3%	C: 8.0 ± 5.8%	C: 8.0 ± 5.8%
E: 7.4 ± 15.3%	E: 4.8 ± 2.8%	E: 4.8 ± 2.8%	E: 6.1 ± 6.0%	E: 6.1 ± 6.0%
Ed: 5.7 ± 11.0%	Ed: 7.2 ± 5.9%	Ed: 7.2 ± 5.9%	Ed: 4.8 ± 4.8%	Ed: 4.8 ± 4.8%
*Actinobacteria*	*Negativicutes*	*Selenomonadales*	*Erysipelotrichaceae*	*Ruminococcus*
				*_gnavus_group*
C: 2.3 ± 1.6%	C: 3.6 ± 3.0%	C: 3.6 ± 3.0%	C: 6.6 ± 3.3%	C: 5.0 ± 6.3%
E: 1.9 ± 1.8%	E: 9.8 ± 15.7%	E: 9.8 ± 15.7%	E: 4.8 ± 2.8%	E: 4.1 ± 2.3%
Ed: 4.0 ± 5.1%	Ed: 7.1 ± 6.8%	Ed: 7.1 ± 6.8%	Ed: 7.2 ± 5.9%	Ed: 4.9 ± 3.5%
*Tenericutes*	*Coriobacteriia*	*Coriobacteriales*	*Prevotellaceae*	*Alloprevotella*
C: 0.001 ± 0.0003%	C: 2.3 ± 1.6%	C: 2.3 ± 1.6%	C: 7.2 ± 5.9%	C: 2.9 ± 3.7%
E: 0.001 ± 0.0003%	E: 1.9 ± 1.8%	E: 1.9 ± 1.8%	E: 3.4 ± 4.4%	E: 2.7 ± 4.0%
Ed: 0.003 ± 0.005%	Ed: 3.9 ± 5.1%	Ed: 3.9 ± 5.1%	Ed: 4.6 ± 5.0%	Ed: 2.0 ± 2.1%
*Deferribacteres*	*Gammaproteobacteria*	*Lactobacillales*	*Veillonellaceae*	*Prevotella_9*
C: 0.003 ± 0.0004%	C: 1.1 ± 0.6%	C: 0.7 ± 1.7%	C: 2.5 ± 3.0%	C: 2.8 ± 3.7%
E: 0.0001 ± 0.0002%	E: 6.5 ± 14.3%	E: 2.0 ± 3.9%	E: 9.1 ± 15.8%	E: 0.5 ± 0.6%
Ed: 0.001 ± 0.003%	Ed: 4.1 ± 8.1%	Ed: 5.2 ± 7.6%	Ed: 6.6 ± 6.7%	Ed: 2.5 ± 3.1%
*Cyanobacteria*	*Bacilli*	*Enterobacteriales*	*Streptococcaceae*	*Megamonas*
C: 0.001 ± 0.004%	C: 0.8 ± 1.7%	C: 0.3 ± 0.2%	C: 0.7 ± 1.7%	C: 2.5 ± 3.0%
E: 0.0 ± 0.0%	E: 2.0 ± 3.9%	E: 6.0 ± 14.4%	E: 1.3 ± 3.4%	E: 9.1 ± 15.7%
Ed: 0.0 ± 0.0%	Ed: 5.2 ± 7.6%	Ed: 3.9 ± 8.2%	Ed: 3.8 ± 7.6%	Ed: 5.5 ± 6.9%
*Thermomicrobia*	*Betaproteobacteria*	*Burkholderiales*	*Enterobacteriaceae*	*Streptococcus*
C: 0.0 ± 0.0%	C: 0.6 ± 0.6%	C: 0.6 ± 0.6%	C: 0.3 ± 0.2%	C: 0.7 ± 1.7%
E: 0.0001 ± 0.003%	E: 0.8 ± 10.1%	E: 0.8 ± 10.1%	E: 6.0 ± 14.4%	E: 1.3 ± 3.4%
Ed: 0.0007 ± 0.0002%	Ed: 1.5 ± 3.0%	Ed: 1.5 ± 3.0%	Ed: 3.9 ± 8.2%	Ed: 3.8 ± 7.6%
*Chloroflexi*	*Unidentified*	*Aeromonadales*	*Lactobacillaceae*	*Lactobacillus*
C: 0.0 ± 0.0%	*Actinobacteria*	C: 0.5 ± 0.4%	C: 0.05 ± 0.1%	C: 0.05 ± 1.2%
E: 0.0 ± 0.0%	C: 0.01 ± 0.01%	E: 0.5 ± 0.5%	E: 0.5 ± 1.0%	E: 0.5 ± 1.0%
Ed: 0.001 ± 0.003%	E: 0.02 ± 0.02%	Ed: 0.2 ± 0.2%	Ed: 0.1 ± 0.2%	Ed: 0.1 ± 0.2%
	Ed: 0.07 ± 0.09%			

## Data Availability

The data of the sequences are available in NCBI Sequence Read Archive (SRA), BioProject ID PRJNA746550.
